# Attention-Based Sentiment Region Importance and Relationship Analysis for Image Sentiment Recognition

**DOI:** 10.1155/2022/9772714

**Published:** 2022-11-17

**Authors:** Shanliang Yang, Linlin Xing, Zheng Chang, Yongming Li

**Affiliations:** ^1^School of Computer Science and Technology, Shandong University of Technology, Zibo 255000, China; ^2^Institute of Scientific and Technical Information, Shandong University of Technology, Zibo 255000, China

## Abstract

Image sentiment recognition has attracted considerable attention from academia and industry due to the increasing tendency of expressing opinions via images and videos online. Previous studies focus on multilevel representation from global and local views to improve recognition performance. However, it is insufficient to research the importance and relationship of visual regions for image sentiment recognition. This paper proposes an attention-based sentiment region importance and relationship (ASRIR) analysis method, including important attention and relation attention for image sentiment recognition. First, we extract spatial region features using a multilevel pyramid network from the image. Second, we design important attention to exploring the sentiment semantic-related regions and relation attention to investigating the relationship between regions. In order to release the excessive concentration of attention, we employ a unimodal function as the objective function for regularization. Finally, the region features weighted by the attention mechanism are fused and input into a fully connected layer for classification. Extensive experiments on various commonly used image sentiment datasets demonstrate that our proposed method outperforms the state-of-the-art approaches.

## 1. Introduction

With the maturity of the multimedia platform, more and more people tend to share their feelings on social media. Netizens increasingly like to use images to express their emotion. In order to understand the opinion and attitudes toward specific events, sentiment recognition is indispensable. Automatically recognizing the sentiment in an image has various applications, such as opinion mining, smart advertising, and entertainment. From the perspective of psychology, human emotion can be evoked by visual elements and sentiment regions [1, 2]. While previous studies focus on multilevel representation from global and local views to improve recognition performance, it is insufficient to research the importance and relationship of visual regions for image sentiment recognition.

Image sentiment recognition is confronted with various challenges due to the abstraction and subjectivity of visual emotion. The sentiment evoked by image content is a much higher level of abstraction [3], bringing about a massive gap between the sentiment category and image content. The same emotion category probably is evoked by multiple regions in an image [4], and interaction occurs between regions in arousing emotion, making the sentiment feature extremely difficult to extract. Early studies address these challenges by designing low-level, middle-level, and high-level features to describe the sentiment information conveyed by the image. With the deep neural network development in computer vision tasks, the deep learning approach has obvious advantages compared with hand-crafted features. Significantly, the convolution neural network can automatically extract the deep representation of the image, which has a hierarchical architecture to learn low-level to high-level features [5–7]. Low-level features indicate color, shape, line, and texture. Middle-level features represent the composition, emphasis, and aesthetics of the image. High-level features express the semantics of the image from the global view.

As in the previous study, emotion regions play a critical role in image sentiment recognition. Firstly, the process of the emotion excited by one image is complex, not only handling global information but also dealing with descriptive local regions. When observing the image, people always first are attracted by the most salient region under the control of the attention mechanism. Notably, different regions in an image have different capacities for evoking emotion. As such, some researchers have studied the importance of emotion regions [3, 8, 9]. Secondly, the evoked emotion has a connection with the interaction between different regions in the image. The relationship between visual regions can produce visual semantics and influence the aroused sentiment. For instance, the image including one lovely girl and beautiful flowers expresses positive sentiment. In comparison, the image consisting of one girl and one ferocious tiger could express the negative sentiment of fear. Previous research emphasized the relationship between the local region and global information or employed multilevel features. However, the importance of visual regions and the relationship between the local regions have not received sufficient attention and research, essential for image sentiment recognition.

**Figure 1:**
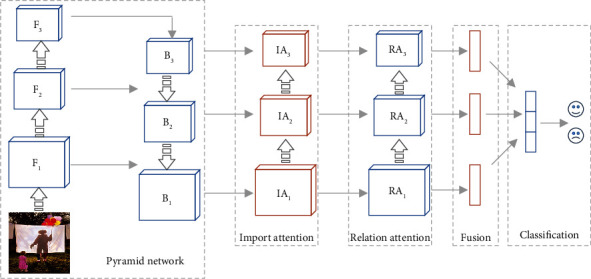
The illustration of our proposed model for image sentiment recognition, which is consisted of five components: pyramid network based on backbone convolution network, sentiment important attention, sentiment relation attention, feature fusion, and classification.

In order to fully explore the sentiment semantic information of visual regions, we propose an attention-based sentiment region importance and relationship (ASRIR) analysis model. An illustration of the proposed model is shown in Figure 1. The pyramid network extracts the global and multilevel feature maps through bottom-up and top-down convolution layers. Then, we design the sentiment important attention to learn the contribution of different regions for sentiment representation and emphasize the sentiment semantic-related regions. We build the sentiment relation attention to learn the interaction of visual regions in arousing sentiment and investigate the relationship between spatial regions. The feature maps weighted by the proposed attention mechanism are integrated into a discriminative representation. Finally, the sentiment representation is input into a fully connected layer to recognize the sentiment category. The main contribution of this work can be summarized as follows.

1. We propose a novel model for image sentiment recognition. The model combines the multilevel features and sentiment semantic information of local regions, which analyzes the importance of regions and the relationship between regions.
2. We design sentiment important attention and sentiment relation attention to learn the sentiment contribution and the semantic relationship of different local regions, which produce the final sentiment representation to improve the performance of image sentiment recognition.
3. We produce extensive experiments on commonly used sentiment datasets. Experimental results show that our proposed framework outperforms the state-of-the-art approaches.

The rest of this paper is organized as follows. Section 2 describes the most relevant research on image sentiment recognition. The proposed model of ASRIR is explained in detail in Section 3. Extensive experiments on public sentiment datasets are conducted to verify the performance of the proposed model in Section 4. Section5 evaluates the experimental results and implements the visual analysis. Section 6 is the conclusion of this work.

## 2. Related Work

Image sentiment recognition has attracted incremental attention from academia and industry. Researchers have conducted a series of valuable and influential works on image sentiment recognition focusing on visual attention mechanism and sentiment region detection. This section reviews the development of image sentiment recognition from several aspects closely related to our work.

### 2.1. Image Sentiment Recognition

Image sentiment recognition researches the sentiment polarity of humans inspired by visual content. The research direction in this field could be divided into low-level feature methods, semantic-level feature methods, and high-level feature methods according to technological development progress.

Based on image processing and psychology theory, low-level feature methods attempt to map the image to sentiment categories. Inspired by psychological experiments, Wang et al. designed three fuzzy histograms for each emotional factor [10]. Machajdik and Hanbury exploited low-level features including color, texture, composition, faces, and skins inspired by psychology and art theories [11]. Zhao et al. proposed the principles-of-art-based features utilizing balance, emphasis, harmony, and others [12]. Sartori et al. investigated how color combinations evoke emotions in an observer and employed art theory to design features and algorithms [13]. Although low-level features could describe the sentiment-interfering factors, the semantic gap between low-level features and high-level sentiments consistently existed due to the complexity, fuzziness, and globalism of visual sentiment.

Semantic-level feature methods take objects, scenes, and other visual content into consideration to alleviate the semantic gap and employ adjective noun pairs (ANPs) to express sentiment features. Borth et al. developed a large-scale adjective noun pair, which consists of 1200 visual sentiment ontologies building a bridge between emotions and visual objects [14]. In order to take advantage of ANP resources, Li et al. computed the image sentiment value by adding the textual sentiment value of the ANP concept detected in the image [15]. Instead of ANPs, other semantic-level features have been explored. Yuan et al. constructed an interpretable image sentiment prediction framework leveraging the mid-level semantic features [16]. Zhao et al. implemented an emotion distribution prediction task integrating sentiment features at different levels, including mid-level generic features and mid-level special features [17].

With the rapid development of deep learning, especially the successful application in computing vision, deep neural networks have been verified to extract high-level descriptive features. The convolution neural network is a perfect architecture for image processing. Campos et al. explored the application of the CNN network in visual sentiment classification and how the model perceives sentiment information [18]. In order to integrate the content information and the style information, Zhang et al. proposed a novel CNN model to learn discriminative representation for image sentiment recognition [19]. Aslan et al. proposed a new artistic knowledge graph to promote the emotion classification system, which exploits visual, contextual, and emotional information [20]. In addition to the CNN network, the recurrent neural network is also investigated in sentiment recognition. Zhu et al. proposed a unified CNN-RNN to exploit the dependency among different feature levels by employing a bidirectional recurrent neural network [21]. The emotion region in the image is critical for sentiment recognition. However, it is not easy to locate the emotional region directly. In order to tackle this problem, Yang et al. proposed a weakly supervised model composed of a sentiment map detection branch and classification branch [22, 23]. The convolution layers of the CNN network express different level feature that is conducive to image sentiment recognition. Rao et al. investigated the multilevel deep representations by combining multiple convolutional neural network layers [5, 24].

### 2.2. Visual Attention Mechanism

Psychologists have found the visual attention mechanism of the human eye. People always focus on a particular portion of the visual field that attracts their attention. The subjective region can stimulate human emotion more than the objective region. Inspired by this theory, a series of research extract sentiment regions using the deep learning model with an attention mechanism. Song et al. proposed visual attention to locate the sentiment areas in an image [25]. Wu et al. developed a multiattention model to discover and localize the sentiment-relevant regions [26]. Yadav and Vishwakarma proposed a residual attention model which includes a trunk branch and a mask branch that learns the importance of different regions [27]. Ragusa et al. utilized a saliency detector to produce salient regions [28].

The most notable characteristic of the convolution layer is a series of channels for extracting diverse feature maps, which have discrepant importance for sentiment recognition. Researchers have investigated channel attention for modeling this phenomenon. Fan et al. designed a channel weighting subnetwork to compute a set of feature weights for every feature map [29]. Self-attention is a crucial method for building relationships between queries and key features, which is beneficial for exploring the association between visual regions. He et al. extracted local visual features by pyramid network and mined the association between local visual features through a self-attention mechanism [30]. Bera et al. extracted semantic regions using SIFT key points and focused on the most relevant regions utilizing attention mechanisms [31]. With the development of visual attention, more and more methods integrate multiple attention mechanisms for image sentiment recognition. Zhao et al. explored the spatial connectivity patterns and interdependency between channels through spatialwise attention and channelwise attention [32]. Li et al. employed spatial attention to enhance the contrast between salient and irrelevant regions and adopted channel attention to emphasize informative features [33]. Ding et al. proposed pyramid spatial attention and pyramid channel attention to locate discriminative regions [34].

### 2.3. Sentiment Region Detection

Not all information in the image is valuable for sentiment recognition. Some regions express more important emotional information than others, attracting people's attention and stimulating emotions. You et al. paid attention to the local area relevant to human emotional response and studied the impact of local regions on visual sentiment recognition [9]. Yang et al. proposed a framework to automatically discover effective regions by computing object and sentiment scores [35]. Xiong et al. designed a region-based convolution neural network to detect sentiment regions automatically and concentrate on essential sentiment factors [36]. Zheng et al. exploited the different contributions of local regions to visual sentiment recognition concerning the global image [37]. Zhang et al. proposed a region attention network to capture the importance of face regions, which embeds a varied number of region features into a fixed-length representation [38]. It is advantageous to improve the performance of sentiment recognition by combining local sentiment regions and global image features. Wu et al. proposed a scheme combining global and local information by fusing subimage with the salient object and entire image [3]. In addition to locating sentiment regions from a global and local view, image sentiment is closely related to different levels of visual features. Rao et al. proposed a multilevel region-based convolution neural network to utilize different levels of sentiment regions [39]. The great majority of the above research focuses on discovering sentiment regions. However, the semantic relationship between regions is critical for sentiment representation. Zhang et al. designed a novel model exploring the relationship between the image sentiment and semantic object combination in an image [40]. Then, they proposed a multilevel correlation analysis model of sentiment regions to exploit the effects of the interactions between sentiment regions [8].

## 3. Image Sentiment Recognition Model

This section introduces the proposed model ASRIR for image sentiment recognition. An overview of the attention-based sentiment region importance and relationship analysis network is shown in Figure 1. Sentiment important attention and sentiment relation attention are adopted to improve performance. Firstly, the pyramid network takes an image as input and extracts region features, including multilevel semantic information. The pyramidal features represent the image regions by the architecture of convolution and pooling. Secondly, we design the important attention and relation attention mechanism based on the pyramidal feature to learn the importance of different regions and the relationship between regions. Finally, once the attention weights have been obtained from the attention mechanism, multilevel discriminative representations weighted by attention are fused and input into a fully connected layer for sentiment recognition. The whole proposed network can be trained end-to-end, and the framework is flexible in the backbone architecture.

### 3.1. Region Feature Extraction

The CNN-style network has a strong capability of extracting visual features. Therefore, we investigate the feature maps extracted by a convolutional neural network and attempt to build the bridge between image features and sentiment polarity. ResNet architecture is the widely accepted model for image processing, which significantly improves the performance of various tasks, such as image classification, object detection, and image segmentation. We employ ResNet as our backbone architecture for producing image representation without loss of generality. The ResNet50 network is pretrained on the ImageNet image recognition dataset, consisting of about 15 million labeled images from 22 thousand different categories [41]. For image *x*, we extract the feature *F* ∈ *ℝ*^*h*×*w*×*c*^ from the convolutional layer, in which *h* and *w* are the height and width of the feature map, and *c* is the number of channels.

**Figure 2:**
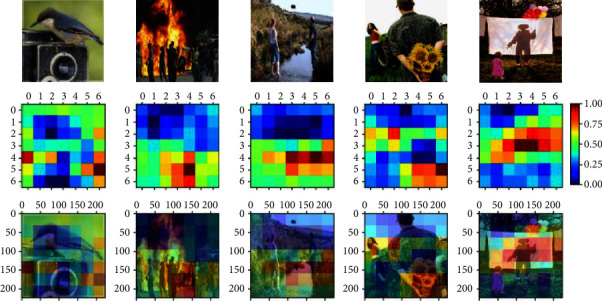
The visualization of the feature map extracted from the Conv5_3 layer in ResNet50. The first row is the original image in experiment datasets expressing various sentiments. The second row is the feature map of the Conv5_3 layer with a 7 × 7 matrix ranging from zero to one by processing the corresponding image. The third row is the artificial color map by integrating the original image and the feature map.

In the extracted feature *F*, *h* and *w* could be regarded as the number of spatial regions determined by the network architecture and the image resolution. The channel number *c* indicates the representation dimension for each region. The region in the image could be denoted as *f*_*i*_ ∈ *ℝ*^*c*^, and the whole feature map could be represented as [*f*_1_, *f*_2_,…, *f*_*hw*_] ∈ *ℝ*^*hw*×*c*^. Image regions with salient object play a critical role in visual sentiment expression. We visualize the feature map of the Conv5_3 layer of ResNet50 as shown in Figure 2. In the second row, every color square represents one spatial region in the image. The value of the feature map from zero to one indicates the contribution to the downstream task. The value of the red color region is close to one, and the value of the blue color region is close to zero. As we can see from the third row, the red color concentrates on the objects in the image, which means that the convolution network can extract significant regions. However, there are still some problems with the extracted regions. For instance, the region's contribution to sentiment representation is difficult to distinguish, and the relationship between regions is challenged to represent only utilizing the feature map. The importance of regions and the relationship between regions are two critical aspects affecting the performance of sentiment recognition. Attention mechanisms allow us to efficiently deal with the limitation of the convolution network by selecting the relevant information and filtering out the irrelevant information. Therefore, we design sentiment important attention and relation attention mechanism for image sentiment recognition.

### 3.2. Sentiment Important Attention

**Figure 3:**
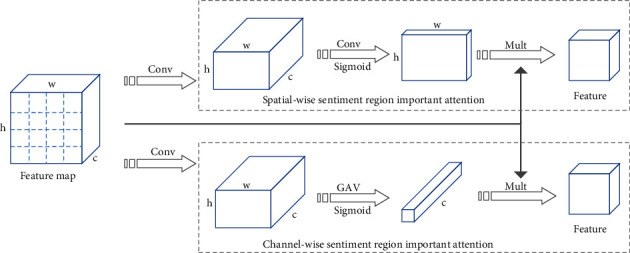
The illustration of sentiment important attention consists of two branches, spatialwise sentiment important attention and channelwise sentiment important attention.

Image region plays a vital role in expressing sentiment. However, the regions in one image have different contributions to predicting sentiment polarity. Therefore, directly employing a global visual feature from a convolution network to predict sentiment may lead to unsatisfactory results due to the irrelevant regions. Considering the feature map has spatial dimension and channel dimension, we design spatialwise sentiment important attention for emphasizing the sentiment semantic-related regions and channelwise sentiment important attention for selecting semantic attributes of different channels. The architecture of sentiment important attention is shown in Figure 3. We introduce the detail of spatialwise important attention and channelwise important attention in the following.

Spatialwise important attention consists of two 1 × 1 convolution layers and a sigmoid function generating the spatial attention distribution over all the image regions. The calculation formula is shown as follows:

(1) $$\begin{array}{c} H_{s}=W_{s1}\left[\tanh\;\left(W_{s2}F+b_{s}\right)\right],\\ {IA}_{s}=Sigmoid\left(H_{s}\right)\text{,} \end{array}$$

where *W*_*s*1_ ∈ *ℝ*^1×*k*^ and *W*_*s*2_ ∈ *ℝ*^*k*×*h*^ are parameter matrices, *k* is the size of the hidden layer, b_s_ ∈ *ℝ*^*k*^ is a *k*-dimension bias vector, tanh is the standard nonlinear hyperbolic tangent function. Accordingly, *IA*_*s*_ ∈ *ℝ*^*h*×*w*^ corresponds to the spatial attention distribution, and the element of the attention matrix indicates the importance of the image region. Then, the weighted feature map based on spatialwise important attention is obtained as follows:

(2) $$\begin{array}{c} \left.F_{SI}={{IA}_{s}⊙\left(W\right)}_{s2}F+b_{s}\right)\text{,} \end{array}$$

where is the multiplication of the feature map and attention matrix, which is performed by multiplying each element of the attention matrix to each image region feature vector.

Channelwise important attention consists of one 1 × 1 convolution layer, one global average pooling, and a sigmoid function generating the channel attention distribution over all the feature channels. The calculation formula is shown as follows:

(3) $$\begin{array}{c} H_{c}=GAP\left(\tanh\;\left(W_{c1}F+b_{c}\right)\right),\\ {IA}_{c}=Sigmoid\left(H_{c}\right)\text{,} \end{array}$$

where GAP is the abbreviation of global average pooling, *W*_*c*1_ ∈ *ℝ*^*k*×*h*^ is the parameter matrix, *b*_*c*_ ∈ *ℝ*^*k*^ is a *k*-dimension bias vector. Accordingly, *IA*_*c*_ ∈ *ℝ*^*c*^ corresponds to the channel attention distribution, and the weight value indicates the contribution of different channels for sentiment recognition. Then, the weighted feature map based on channelwise attention is obtained as follows:

(4) $$\begin{array}{c} \left.F_{CI}={{IA}_{c}⊙\left(W\right)}_{c1}F+b_{c}\right)\text{,} \end{array}$$

where ⊙ is the linear combination between the feature map and attention weight vector, which is performed by multiplying the element of attention vector to each corresponding raw of the feature map.

### 3.3. Sentiment Relation Attention

**Figure 4:**
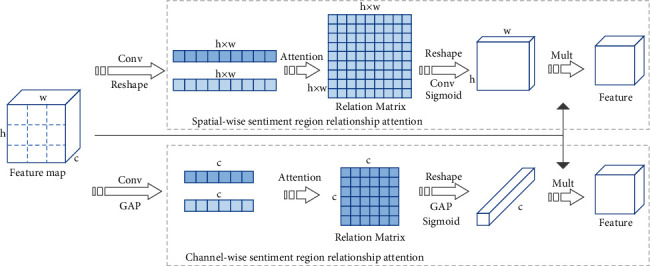
The illustration of sentiment relation attention consists of two branches, spatialwise sentiment relation attention and channelwise sentiment relation attention.

In addition to the importance of each region, the relationship between image regions also plays a critical role in recognizing visual sentiment. To explore the semantic relationship between regions, we design sentiment relation attention which consists of spatialwise sentiment relation attention for investigating the relationship between spatial regions and channelwise sentiment relation attention for analyzing the semantic relation between channels. The architecture of sentiment relation attention is shown in Figure 4. The following is the detail of spatialwise relation attention and channelwise relation attention.

Spatialwise relation attention explores the relationship between different regions. The region feature vector is obtained by one 1 × 1 convolution layer and one reshape operation, which is calculated as follows:

(5) $$\begin{array}{c} \begin{array}{c} V_{s1}=Rashape\left(\tanh\;\left(W_{s1}F+b_{s1}\right)\right),\\ V_{s2}=Rashape\left(\tanh\;\left(W_{s2}F+b_{s2}\right)\right), \end{array} \end{array}$$

where *W*_*s*1_ ∈ *ℝ*^*k*×*h*^ and *W*_*s*1_ ∈ *ℝ*^*k*×*h*^ are parameter matrices, *k* is the size of the hidden layer, *b*_*s*1_ ∈ *ℝ*^*k*^ and *b*_*s*1_ ∈ *ℝ*^*k*^ are bias vectors, *V*_*s*1_ ∈ *ℝ*^*n*×*k*^ and *V*_*s*2_ ∈ *ℝ*^*n*×*k*^ are region features of image, *n* is the number of regions and also indicates *h* × *w*. We employ dot multiplication to obtain the spatial relation matrix as follows:

(6) $$\begin{array}{c} R_{s}=V_{s1}∙{V_{s2}}^{T}\text{,} \end{array}$$

where *R*_*s*_ ∈ *ℝ*^*n*×*n*^ is the relation matrix, and *r*_*i*,*j*_^*s*^ is the element of the relation matrix indicating the relationship between region *i* and region *j*. Then, we obtain an attention map by the reshape operation, convolution layer, and sigmoid activation function. The calculation formula is shown as follows:

(7) $$\begin{array}{c} {RA}_{s}=Sigmoid\left(W_{s3}Reshape\left(R_{s}\right)+b_{s3}\right)\text{,} \end{array}$$

where *W*_*s*3_ ∈ *ℝ*^1×*h*^ is the parameter matrix, *b*_*s*3_ ∈ *ℝ*^*h*^ is the bias vector, and *RA*_*s*_ ∈ *ℝ*^1×*h*×*w*^ is the attention weight of spatial relation attention. Then, the weighted feature map based on spatialwise relation attention is obtained as follows:

(8) $$\begin{array}{c} \left.F_{SR}={{RA}_{s}⊙\left(W\right)}_{s4}F+b_{s4}\right)\text{.} \end{array}$$

Channelwise relation attention investigates the relationship between different channels of the feature map. The channel feature vector is obtained by one 1 ×1 convolution and a reshape operation, which can be expressed as follows:

(9) $$\begin{array}{c} \begin{array}{c} V_{c1}=Rashape\left(\tanh\;\left(W_{c1}F+b_{c1}\right)\right),\\ V_{c2}=Rashape\left(\tanh\;\left(W_{c2}F+b_{c2}\right)\right), \end{array} \end{array}$$

where *W*_*c*1_ ∈ *ℝ*^k×h^ and *W*_*c*2_ ∈ *ℝ*^k×h^ are two parameter matrices, *k* is the size of the hidden layer, *b*_*c*1_ ∈ *ℝ*^*k*^ and *b*_*c*1_ ∈ *ℝ*^*k*^ are bias vectors, *V*_*c*1_ ∈ *ℝ*^*c*×*k*^ and *V*_*c*2_ ∈ *ℝ*^*c*×*k*^ are channel features of the image. We employ dot multiplication to obtain the channel relation matrix as follows:

(10) $$\begin{array}{c} R_{c}=V_{c1}∙{V_{c2}}^{T}\text{,} \end{array}$$

where *R*_*c*_ ∈ *ℝ*^*c*×*c*^ is the relation matrix, and *r*_*i*,*j*_^*c*^ is the element of the matrix indicating the relationship between channel *i* and channel *j*. Then, we obtain an attention map by the reshape operation, global average pooling, and sigmoid activation function, which is shown as follows:

(11) $$\begin{array}{c} {RA}_{c}=Sigmoid\left(GAP\left(Reshape\left(R_{c}\right)+b_{c3}\right)\right)\text{,} \end{array}$$

where GAP is the abbreviation of global average pooling and *RA*_*c*_ ∈ *ℝ*^1×1×*c*^ is the attention weight of channel relation attention. Then, the weighted feature map based on channelwise relation attention is obtained as follows:

(12) $$\begin{array}{c} \left.F_{CR}={{RA}_{c}⊙\left(W\right)}_{c4}F+b_{c4}\right)\text{.} \end{array}$$

### 3.4. Multilevel Pyramid Network

As a general rule, the deep learning model consists of numerous layers from bottom to up. The features extracted from different layers represent different semantic information. Low-level visual features, middle-level image aesthetics, and high-level image semantics could be extracted from different level layers in the CNN style model [5, 42]. In order to extract multilevel feature representation for visual sentiment recognition, we employ the framework of the feature pyramid network (FPN) [43]. The architecture of the multilevel pyramid network in our proposed model is shown in Figure 1, which consists of two critical parts, a bottom-up pathway, and a top-down pathway.

The bottom-up pathway generates the hierarchical feature maps in different sizes using a backbone network, such as ResNet101. We defined the output of each bottleneck in the backbone network as the feature maps to represent the different level semantic information. The feature maps are marked as *B*_1_, *B*_2_, and *B*_3_ for the output of three bottlenecks, conv3, conv4, and conv5. In order to integrate high-level semantic information and low-level spatial information, the top-down pathway is used to combine the feature maps extracted from the bottom-up pathway. The top-down pathway firstly up-samples the higher-level feature map and then merges the up-sampled map with the corresponding bottom-up feature map by element-wise addition. The final feature maps of the multilevel pyramid network are marked as *F*_1_, *F*_2_, and *F*_3_. The pyramidal features can locate samples on different levels and focus on subtle differences of image regions from different scales, benefiting image sentiment recognition.

### 3.5. Feature Fusion

In order to take great advantage of semantic information from sentiment important attention and relation attention, we employ two feature fusion methods, horizontal fusion and vertical fusion. Horizontal fusion integrates the feature maps calculated by sentiment important attention and sentiment relation attention at the same pyramid layer, which is implemented by concatenation operation generating three feature maps *O*_1_, *O*_2_, and *O*_3_. Vertical fusion combines the feature maps from different pyramid layers. Due to the inconsistent dimension of different level feature maps, we utilize subsampling to uniform the feature size. Then, we employ an element-wise addition function to merge the information from different layers, which is shown as follows:

(13) $$\begin{array}{c} O_{i}=\left[F_{SI},F_{CI},F_{SR},F_{CR}\right]\text{,} \end{array}$$

(14) $$\begin{array}{c} O=f\left(O_{1},O_{2},O_{3}\right)\text{.} \end{array}$$

### 3.6. Overall Objective

The overall objective function comprises the classification objective function and the regularization objective function. We combine the two objective functions in the same proportion and optimize the overall objective function by adaptive moment estimation (Adam).

(15) $$\begin{array}{c} L=L_{cls}+L_{reg}\text{.} \end{array}$$

We use a global average pooling and a fully connected layer to generate the discriminative feature vector for sentiment recognition. Followed by a softmax layer, the feature vector is transformed into the probability distribution of sentiment categories. We select the category with the highest probability as the predicted sentiment of the input image. The objective function of classification is

(16) $$\begin{array}{c} L_{cls}=\underset{\left(x,y\right)\in D}{\sum }\underset{c\in C}{\sum }y^{c}\;\log\;f^{c}\left(x;\theta \right)\text{,} \end{array}$$

where *y* is the golden label of image sentiment, *x* is the input image sample, *f* is the proposed model that outputs the corresponding probability of each category, *c* is the sentiment category, *θ* is the set of parameters, and *D* is the training dataset.

The attention weights of important attention and relation attention have excessive concentration leading to losing critical information for sentiment recognition. In order to relieve this problem, we retain as much critical information by controlling the variance of the attention weight. In general, the attention weight contains more information when the variance is greater. Therefore, we employ a unimodal function of the average of all attention weight variances as a regularization objective function:

(17) $$\begin{array}{c} \left.\left.{D=Avg\backslash \backslash \left(\sigma \right.\left(I\right.A}_{s}\right)+\sigma \left({IA}_{c}\right)+\sigma \left({RA}_{s}\right)+\sigma \left({RA}_{c}\right)\right)\text{,}\\ L_{reg}=D+\frac{b}{D}\text{,} \end{array}$$

where *σ* indicates the variance function of the attention weight, Avg is the abbreviation of the average function, and *b* is the hyperparameter to control the function. The objective function encourages the variance to be $\sqrt{b}$, which is an empirical image variance value of the training datasets. We will find the appropriate parameter through experiments.

## 4. Experimental Results

### 4.1. Datasets

We conduct the experiments on six benchmark datasets for image sentiment recognition, including Abstract, IAPSa, Artphoto, TwitterLDL, FlickrLDL, and FI (Flickr and Instagram). These datasets are annotated using the Mikels emotion model [1] with eight sentiment categories. The statistic of these datasets is shown in Table 1, and a brief introduction of them is as follows.

#### 4.1.1. Abstract

The dataset consists of 228 images combining color and texture [2]. About 230 people annotated these images by selecting the best-fitting emotional categories. The ground truth is the category that obtains the most votes without any indeterminism.

#### 4.1.2. IAPSa

The dataset is a subset of the International Affective Picture System (IAPS), which is an emotional image dataset widely used in the investigation of emotion and attention and contains 1,182 images with various contents [1]. IAPSa selects 209 negative images and 186 positive images from the IAPS dataset and labels these images with eight sentiment categories.

#### 4.1.3. Artphoto

The dataset includes 806 images selected from the art-sharing site using emotion categories as search terms [2]. The artist who uploaded the image determined the sentiment category evoked by the conscious manipulation of the emotional objects, lighting, colors, etc.

#### 4.1.4. TwitterLDL

The dataset is collected from Twitter by searching various sentiment keywords [44, 45]. This dataset contains 10045 images after deduplication. A total of 8 annotators were employed to annotate these images with Mikel's eight sentiment categories.

#### 4.1.5. FlickrLDL

The dataset is a subset of the Flickr dataset for learning visual sentiment distribution [44, 45]. This dataset contains 11150 images. Eleven annotators were employed to label these images with Mikel's eight sentiment categories based on their emotional reactions.

**FI** dataset is well known as the large labeled image sentiment dataset, which is collected from two social networks, Flickr and Instagram [46]. Two hundred and twenty-five workers from AMT were employed to annotate the dataset with eight sentiment categories. Finally, 23308 images are well labeled with at least three agreements.

### 4.2. Implementation Details

We implement the proposed model with TensorFlow and Keras framework. The backbone network ResNet101 was pretrained on ImageNet [47]. We trained the models using Adaptive moment estimation (Adam) [48] for 100 epochs on GPU. In the big datasets, FI, TwitterLDL, and FlikrLDL, the batch size was set to 64. The learning rate was initialized to 0.0001 and reduced by 10 every 10 epochs. in the small-scale datasets, Abstract, IAPSa, and Art photo, the batch size was set to 32. The learning rate was initialized to 0.001, and the decay strategy is the same as above. The training images were resized to 256 × 256 and randomly cropped into a 224 × 224 sub-image. We employed data augmentation techniques, such as random horizontal flipping and random cutout. These preprocess methods can help avoid overfitting problems and improve generalization ability. The image data was normalized to zero and one before inputting the network. In the test stage, we resized the image and randomly cropped it into a sub-image. We run the model on the dataset three times and calculate the average result as the recognition performance.

### 4.3. Baseline

This section will introduce the baseline methods for comparing with the proposed model. Due to the deep learning method having an apparent advantage, we select a certain amount of high-level feature-based models as baseline methods.

#### 4.3.1. AlexNet [49]

This network consists of five convolution layers followed by a max-pooling layer, three fully connected layers, and a softmax classifier. We fine-tuned the weight based on ImageNet's pretrained model.

#### 4.3.2. VGGNet16 [50]

This network is a deep convolutional neural network that constructs a framework of 16 layers using small convolution filters. We fine-tuned the model weight based on ImageNet's pretrained model.

#### 4.3.3. PCNN [51]

PCNN is a novel progressive CNN architecture network that firstly trains the model on Flickr images and further fine-tunes the trained model using selected train samples.

#### 4.3.4. ResNet [52]

ResNet eases the training of the deep neural network by using a residual learning framework. In the experiment, we employ two version models pretrained on ImageNet, ResNet50, and ResNet101.

#### 4.3.5. ViT [53]

A transformer is based on the model for image classification, which takes the sequence of image patches as input.

#### 4.3.6. Zhu [21]

A unified CNN-RNN model predicts the visual emotion using different level features and their relationship.

#### 4.3.7. Yang [35]

A framework leverages effective regions by considering the object and sentiment scores.

#### 4.3.8. Rao [39]

A multilevel region-based CNN framework utilizes different levels of visual features from both global and local views for image sentiment recognition.

#### 4.3.9. Zhang [4]

An end-to-end deep neural network leverages emotion-intensity learning for image emotion recognition.

### 4.4. Recognition Results

In order to demonstrate the effectiveness of the proposed model, we design experiments performed on the datasets Artphoto and FI for two categories and eight categories recognition. We convert eight sentiment categories to two categories labeling amusement, awe, contentment, and excitement as positive and anger, disgust, fear, and sadness as negative. We analyze the binary sentiment recognition results to compare our model with three popular CNN-style networks. We investigate the confusion matrices of eight sentiment categories to verify the performance of the proposed model.

Table 2 shows the result on the dataset Artphoto. We employ four evaluation metrics, precision, recall, F1, and accuracy. As shown in Table 2, the accuracy of our proposed method reaches 80.86%. Compared to the baseline model ResNet101, our method with important attention improves the performance by 5.80% for accuracy, indicating the effectiveness of sentiment important mechanism, and our method with relation attention for sentiment recognition improves the performance by 6.41% for accuracy, demonstrating the relation attention can model the relationship between local regions. Our method with sentiment important attention and relation attention almost outperforms all other methods in terms of the four metrics of each sentiment category.

Table 3 shows the result on the dataset FI for two categories. Overall, our proposed method consistently outperforms others. In particular, the *F*1 score for the positive and negative categories can achieve 91.77% and 91.21%, which improves the backbone network ResNet101 by 6.86% and 4.08%. Similar to the Artphoto dataset result, the models that recognize sentiment with important attention and relation attention, in general, perform better than the baseline models, which again demonstrates the advantage of the sentiment important attention and sentiment relation attention.

### 4.5. Comparison with the State-of-the-Art Methods

We compare our proposed model with the previous state-of-the-art methods on the six datasets mentioned above (Abstract, IAPSa, Artphoto, TwitterLDL, FlickrLDL, and FI). We train and test the model on each dataset for the large-scale datasets (TwitterLDL, FlickrLDL, and FI). For the small-scale datasets (Abstract, IAPSa, and Artphoto), we first train the model on dataset FI and then transfer the trained model to small datasets by fine-tuning. For comparison, we conduct experiments on the sentiment binary classification task.  Table 4 shows the recognition accuracy and the comparison to several previous methods. Obvious of the results, we can find that the performance of AlexNet is not excellent, which indicates that it is challenging to grasp discriminative features by utilizing a relatively shallow convolution network. With the deeper network and residual structure, the famous CNN style architectures, VGGNet16, ResNet50, and ResNet101, significantly improve recognition performance. However, it is not easy to further improve the performance when the network reaches a certain depth. The PCNN architecture with a progressive training strategy has similar results to other popular methods. ViT [53] applies the transformer method into image classification, which achieves impressive performance by exploring the relationship between image patches. The methods by Zhu [21], Yang [35], Rao [39], and Zhang [4] utilize the deep multilevel features extracted from different levels of CNN architecture and sentiment regions discovered from the image feature map, which achieve relatively high performance. Our method employs the important attention and relation attention of sentiment regions. The accuracy of our method outperforms previous methods on almost all datasets. We achieve 1.38% and 0.55% higher accuracy than Zhang et al.'s method on Artphoto and FI. The results demonstrate the effectiveness and superiority of the proposed method for image sentiment recognition.

## 5. Evaluation

### 5.1. Confusion Matrices on Artphoto

**Figure 5:**
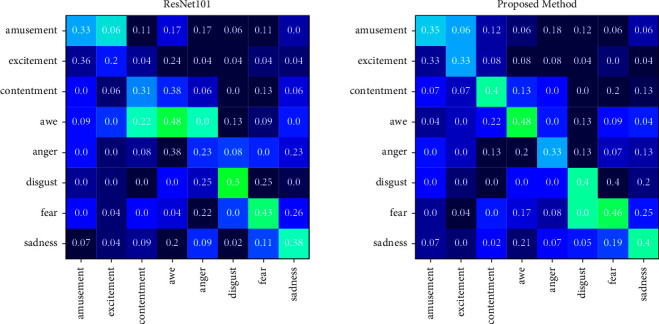
Confusion matrices of ResNet101 and the proposed method experimented on dataset artphoto.

The confusion matrices on dataset Artphoto obtained with the proposed method and baseline network ResNet101 are shown in Figure 5. The recognition accuracy of each category is not more than 50%, possibly because the dataset does not have enough training samples. Our proposed method achieves higher harmonious accuracy for almost all categories than ResNet101. The sentiment categories are most commonly confused with amusement, contentment, and fear. The proposed method achieves more than 40% recognition accuracy for contentment, awe, disgust, fear, and sadness.

### 5.2. Confusion Matrices on FI

**Figure 6:**
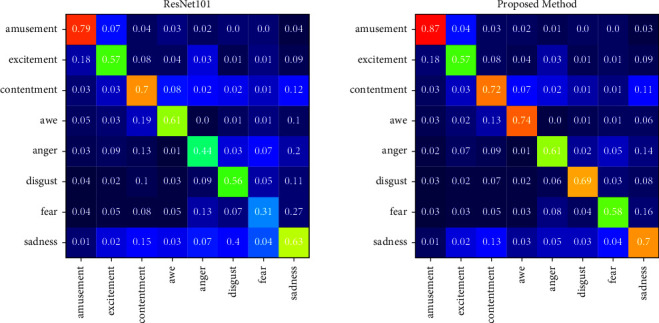
Confusion matrices of ResNet101 and the proposed method experimented on dataset FI.

Figure 6 shows the confusion matrices on dataset FI obtained with the proposed method and the baseline network ResNet101. Compared to the dataset Artphoto, the accuracy of each category improves significantly due to the relatively sufficient training samples. The sentiment categories are most commonly confused with contentment and sadness. The proposed method performs not particularly well for excitement and fear and achieves at least 60% accuracy for the remaining sentiment categories. Applying sentiment important attention and relation attention can improve the performance for image sentiment recognition and obtain a more balanced recognition accuracy for each sentiment category.

### 5.3. The Effect of Parameter *b*

**Figure 7:**
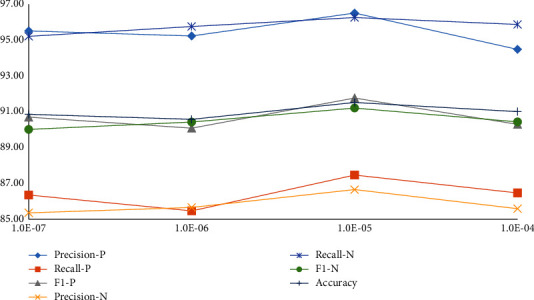
The effect of parameter *b* in our proposed method on the FI dataset. −*P*: the performance of the positive category; −*N*: the performance of the negative category.

The parameter *b* controls the variance of attention weights to retain as much critical information as possible.  Figure 7 depicts the performance curves of our proposed method against various choices of parameter *b* (1.0*E*-7, 1.0*E*-6, 1.0*E*-5, 1.0*E*-4) for investigating the relationship between the performance and parameter *b*. We can see that the accuracy of sentiment recognition shows an upward trend and then a downward trend when *b* varies in a range from 1.0*E*-7 to 1.0*E*-4. The peak performance is achieved when parameter *b* is 1.0*E*-5. Therefore, we choose *b* = 1.0*E*-5 in our experiments to comprehensively control attention distribution.

### 5.4. Visualization

**Figure 8:**
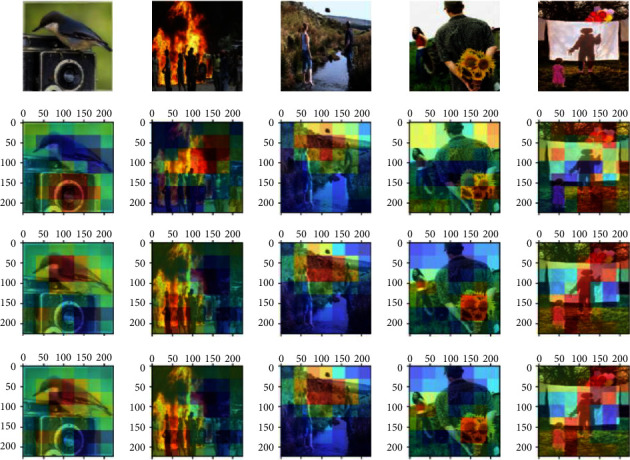
Samples of weighted feature maps produced by attention mechanism. The first row is the original image. The second row is the feature map of the conv5_3 layer in ResNet101. The third row is the feature map weighted by sentiment important attention. The fourth row is the feature map weighted by sentiment relation attention.

To further verify the effectiveness of the proposed attention mechanism, we visualize the weighted feature maps using the heat map generated by the Grad-Cam algorithm [54] and compare them with the original feature map.  Figure 8 shows the feature maps produced by sentiment important attention and sentiment relation attention based on the conv5_3 layer of ResNet101. As illustrated in Figure 8, we observe that the feature maps generated by the convolution layer always ignore the critical information for sentiment recognition, such as important regions, multiple objects, and the relationship between important objects. The feature maps generated by the proposed attention mechanism can focus better on the discriminative regions than the original convolution feature maps. Taking the fifth sample as an example, the important attention map and relation attention map focus on the little girl, the adult, and the balloon, while the original convolution feature map ignores the little girl. Thus, feature maps weighted by important attention and relation attention can extract discriminative features for image sentiment recognition.

## 6. Conclusion

In this paper, we investigate the problem of image sentiment recognition. Inspired by the observation that local regions have different importance for sentiment response and that the relationship between regions contributes much to visual sentiment, we propose a framework to automatically analyze the importance and relationship of regions on multilevel deep feature maps. We extract the multilevel sentiment regions based on the backbone network ResNet101 and combine the multilevel features through the pyramid network. Considering the complexity of the regions evoking sentiment, we design the sentiment important attention and the sentiment relation attention to analyze the regions for image sentiment recognition. Experiment results on various commonly used datasets demonstrate that the proposed framework can achieve excellent performance and outperform other start-of-the-art methods.

## Figures and Tables

**Table 1:** The statistics of the six datasets for experiments of image sentiment recognition.

Dataset	Positive				Negative				Sum
	Amusement	Awe	Contentment	Excitement	Anger	Disgust	Fear	Sadness	
Abstract	25	15	63	36	3	18	36	32	228
IAPSa	37	54	63	55	8	74	42	62	395
Artphoto	101	102	70	105	77	70	115	166	806
TwitterLDL	923	264	7280	714	205	186	241	232	10045
FlickrLDL	1147	1402	6150	503	183	450	580	735	11150
FI	4942	3151	5374	2963	1266	1658	1032	2922	23308

**Table 2:** The result of binary sentiment recognition on abstract dataset.

Model	Positive			Negative			Accuracy (%)
	Precision (%)	Recall (%)	*F*1 (%)	Precision (%)	Recall (%)	*F*1 (%)	
VGGNet16	70.83	66.82	68.77	71.58	71.92	71.75	70.26
ResNet50	75.86	69.62	72.61	72.21	70.14	71.16	71.88
ResNet101	75.01	70.27	72.56	73.02	72.24	72.63	72.60
Ours + important attention	74.83	70.82	72.77	80.58	80.92	80.75	78.40
Ours + relation attention	75.21	74.55	74.88	80.14	81.31	80.72	79.01
Ours + all	80.52	79.49	80.00	81.18	82.14	81.66	80.86

**Table 3:** The result of binary sentiment recognition on the FI dataset.

Model	Positive			Negative			Accuracy (%)
	Precision (%)	Recall (%)	*F*1 (%)	Precision (%)	Recall (%)	*F*1 (%)	
VGGNet16	87.11	79.69	83.23	79.41	86.34	82.73	82.98
ResNet50	88.32	81.43	84.74	83.61	90.25	86.80	85.77
ResNet101	89.48	80.79	84.91	84.04	90.46	87.13	86.02
Ours + important attention	95.27	86.07	90.44	86.22	95.38	90.57	90.50
Ours + relation attention	95.11	87.16	90.96	85.32	95.82	90.27	90.61
Ours + all	96.51	87.47	91.77	86.66	96.26	91.21	91.52

**Table 4:** The results of recognition accuracy by using the proposed network, and the comparison with previous work. The results of previous work employ the estimation in related literature. The symbol ‘—' expresses that there are no appropriate results.

Model	Abstract (%)	IAPSa (%)	Artphoto (%)	TwitterLDL (%)	FlickrLDL (%)	FI (%)
AlexNet [49]	65.49	84.58	69.27	85.10	76.49	72.43
VGGNet16 [50]	72.48	88.51	70.09	90.77	80.53	83.05
PCNN [51]	70.84	88.84	70.96	91.87	82.07	75.34
ResNet50 [52]	73.07	89.95	70.93	91.48	81.61	85.43
ResNet101 [52]	73.36	90.13	71.08	91.68	82.08	85.92
ViT [53]	75.34	89.69	76.52	91.55	83.12	86.33
Zhu [21]	73.88	91.38	75.50	—	—	84.26
Yang [35]	76.03	92.39	74.80	—	—	86.35
Rao [39]	77.77	93.66	77.28	—	—	87.51
Zhang [4]	83.02	95.84	79.24	—	—	90.97
Ours	83.43	94.85	80.62	93.92	85.66	91.52

## Data Availability

The data used to support the findings of this study are included within the article.
